# Quantitative analysis of the *Brucella suis* proteome reveals metabolic adaptation to long-term nutrient starvation

**DOI:** 10.1186/1471-2180-13-199

**Published:** 2013-09-04

**Authors:** Sascha Al Dahouk, Véronique Jubier-Maurin, Heinrich Neubauer, Stephan Köhler

**Affiliations:** 1 Federal Institute for Risk Assessment, Diedersdorfer Weg 1, D-12277, Berlin Germany; 2 Department of Internal Medicine III, RWTH Aachen University, Pauwelsstraße 30, D-52074, Aachen Germany; 3 Université Montpellier I, Centre d’études d’agents Pathogènes et Biotechnologie pour la Santé (CPBS), Montpellier, France; 4 CNRS, UMR 5236, CPBS, F-34293, Montpellier, France; 5 Université Montpellier II, CPBS, F-34095, Montpellier, France; 6 Friedrich-Loeffler-Institut, National Reference Laboratories for Brucella spec. Infections, Naumburgerstraβe 96a, D-07743, Jena, Germany

**Keywords:** *Brucella*, Persistence, Proteome, Starvation, 2D-DIGE

## Abstract

**Background:**

During the infection process, bacteria are confronted with various stress factors including nutrient starvation. In an *in vitro* model, adaptation strategies of nutrient-starved brucellae, which are facultative intracellular pathogens capable of long-term persistence, were determined.

**Results:**

Long-term nutrient starvation in a medium devoid of carbon and nitrogen sources resulted in a rapid decline in viability of *Brucella suis* during the first three weeks, followed by stabilization of the number of viable bacteria for a period of at least three weeks thereafter. A 2D-Difference Gel Electrophoresis (DIGE) approach allowed the characterization of the bacterial proteome under these conditions. A total of 30 proteins showing altered concentrations in comparison with bacteria grown to early stationary phase in rich medium were identified. More than half of the 27 significantly regulated proteins were involved in bacterial metabolism with a marked reduction of the concentrations of enzymes participating in amino acid and nucleic acid biosynthesis. A total of 70% of the significantly regulated proteins showed an increased expression, including proteins involved in the adaptation to harsh conditions, in regulation, and in transport.

**Conclusions:**

The adaptive response of *Brucella suis* most likely contributes to the long-term survival of the pathogen under starvation conditions, and may play a key role in persistence.

## Background

*Brucella* spp. are highly infectious pathogens causing a systemic multi-organ disease in humans and sterility and abortion in animals. Brucellosis is currently the most important bacterial zoonosis worldwide. In the absence of an adequate long-term antibiotic treatment, acute human brucellosis (Malta fever) may relapse or turn into chronic disease [1,2].

During the acute phase of infection, brucellae are capable of replicating in the macrophages of the mammal host where they are found within a nutrient-poor vacuole. Several genes encoding enzymes participating in amino acid and purine or pyrimidine biosynthesis have proven to be essential for intracellular replication [3,4]. At a later stage of chronic infection, persistence of *Brucella* has been evidenced by the detection of live bacteria in abscesses of patients. These bacterial cells could be reactivated to full virulence only by the infection of tissue cultures [5]. The mechanisms enabling *Brucella* to persist in eukaryotic hosts are still unknown. Work on *Mycobacterium tuberculosis* has demonstrated that hypoxia and starvation are key factors triggering bacterial persistence [6]. A starvation model incubating bacteria for several weeks in phosphate-buffered saline and developed 80 years ago [7,8] was chosen for transcriptome and proteome analysis of *M*. *tuberculosis*[9]. Microarray-based analysis confirmed the results obtained by proteomics: the level of transcription, the biosynthesis of lipids and the process of cell division are reduced, whereas several factors involved in long-term survival and in stringent control are induced. To date, only a few *Brucella* proteomic studies have been published which investigated the protein profiles of *B*. *melitensis* grown in rich culture medium [10] or under stress conditions [11], of the cell envelope of *B*. *abortus*[12], and, more recently, of *B*. *suis* during macrophage infection and under oxygen depletion [13,14] and of *B*. *abortus* in macrophages [15].

In addition, viable brucellae are able to persist in the environment, and periods of survival in soil, manure and water have been determined, reaching up to 180, 240, and 150 days, respectively [16]. Soil may even be the natural habitat of the lately described species *B*. *microti*[17].

The aim of our study is to better understand and characterize the adaptation of *B*. *suis* to extreme nutrient starvation as it may occur under specific conditions of persistence during the infection of the host, using a well-described model. A quantitative proteome analysis comparing the protein profiles of brucellae under starvation with those cultured in rich medium was performed.

## Results and discussion

### Survival of *B*. *suis* under extreme starvation conditions

**Figure 1:**
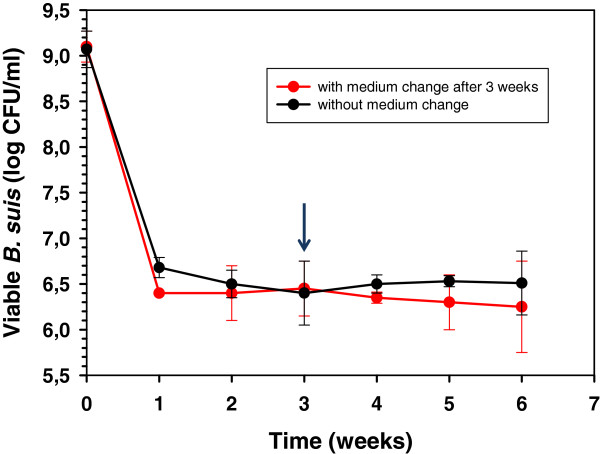
**Survival kinetics of *Brucella *under starvation conditions.** Survival kinetics of *Brucella suis* 1330 in a salt solution over a period of six weeks at 37°C (see Methods Section) with (red curve) or without (black curve) medium change after three weeks of incubation (marked by an arrow). Each graph represents the mean of three independent experiments ± standard deviation.

Based on early work performed on *M*. *tuberculosis*[8], we have developed a simple nutrition starvation model to study the impact on long-term viability of the pathogen. Following growth in rich medium, bacteria were incubated in a salt solution devoid of carbon and nitrogen sources under shaking and aeration. Oxygen concentration was kept constant in order to avoid variation of a second parameter. A sharp decline of approximately 2.5 logs was observed over a period of 2 weeks, followed by stabilisation of the number of viable bacteria during the next 4 weeks (Figure 1). The colony formation on TS solid medium of bacteria sampled from the salt solution for enumeration of viable bacteria confirmed that these maintained their capacity to grow in rich medium. Additional experiments performed under the same conditions but over a period of 27 weeks showed that stable concentrations of viable brucellae were obtained throughout a period of more than 6 months (data not shown). This behaviour indicated the adaptation of a subpopulation of the pathogen to the environmental conditions encountered. The growth curves of *B*. *suis* under nutrient starvation are similar to those of *Mycobacterium* sp. [8,18,19]. Both, long-term survival of a “starvation-resistant” subpopulation and an equilibrium between dying bacteria and those replicating while feeding on nutrients released by dead brucellae, have to be taken into consideration. Washing of the bacteria and replacement of medium after three weeks of incubation, however, did not alter the survival kinetics (Figure 1, red curve), indicating that soluble metabolites originating from dead bacteria may play, at best, a minor role. The lack of net replication of *B*. *suis* is an indirect proof of extreme starvation and indicates the set-up of a state of persistence.

### Proteome analysis of *B*. *suis* after six weeks of nutrient starvation

**Figure 2:**
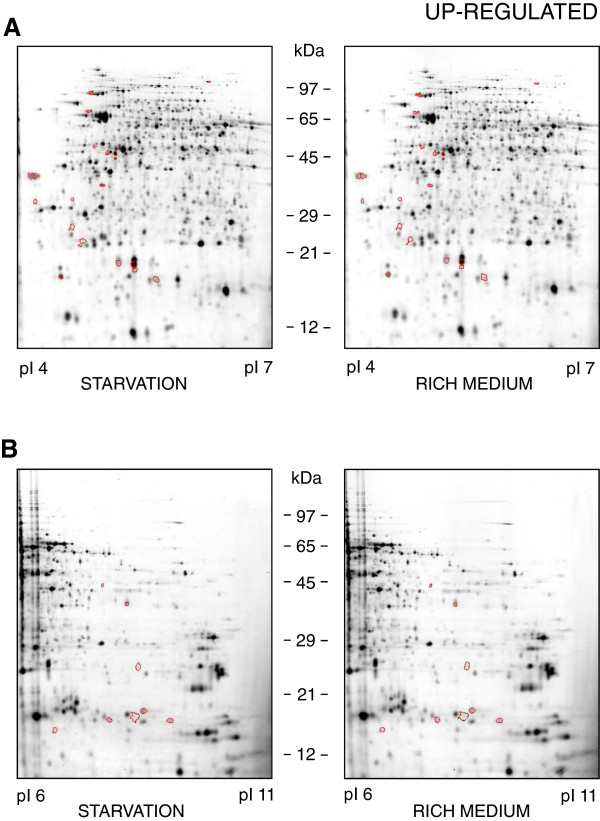
**Up-regulated proteins of *Brucella *under starvation conditions.** Protein profiles of *B*. *suis* 1330 after six weeks under starvation conditions in a salt solution (left panels), or during early stationary phase in TS broth (right panels). Proteins with a pI 4–7 are shown in **(A)**, those with a pI 6–11 in **(B)**. Proteins up-regulated during starvation are encircled.

**Figure 3:**
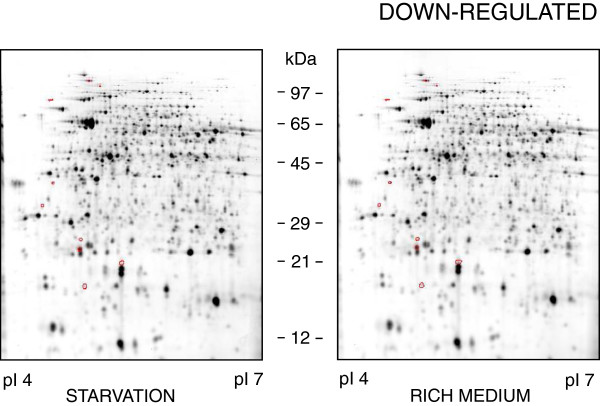
**Down-regulated proteins of *Brucella *under starvation conditions.** Protein profiles of *B*. *suis* 1330 after six weeks under starvation conditions in a salt solution (left panel), or during early stationary phase in TS broth (right panel). Proteins down-regulated during starvation are encircled. Only proteins with pI 4–7 are shown, as no down-regulated proteins with pI 6–11 were detected.

Figures 2 and 3 each show one representative gel out of three featuring the proteomes of *B*. *suis* under long-term starvation conditions (left panels) versus late log/early stationary phase in rich medium (right panels). On the 2D-DIGE reference gels, a total of 2553 and 2284 different protein spots were detected in the pI ranges 4–7 and 6–11, respectively.

Up- and down-regulated proteins during starvation are separately marked in Figures 2 and 3, respectively. Details of these gels together with the tags identifying the spots of interest are available in the Additional files 1 and 2. The proteins with either increasing or decreasing concentrations under long-term starvation are presented in Table 1 and have been classified according to their potential functions.

**Table 1:** **Up- or down-regulated *Brucella suis *proteins under nutrient starvation conditions**

**Spot ID**^**a**^	**ORF**^**b**^	**Protein function**^**c**^	**Theoret.M**_**r**_**/pI**^**d**^	**Fold change**^**e**^	**t-Test**^**f**^
		**Adaptation to atypical conditions**			
2146	BR2149	Dps family protein (DNA-binding proteins from starved cells)	18.2/5.3	2.63	0.00019
429	BR0685	organic solvent tolerance, putative	88.7/5.4	1.53	0.024
2122	BR2149	Dps family protein	18.2/5.3	1.52	0.006
438	BR0685	organic solvent tolerance, putative	88.7/5.3	1.49	0.0004
		**Stress proteins**/**chaperones**, **protein folding**			
1624	BR0171	heat shock protein GrpE	25.2/4.7	−1.42	0.039
662	BR2125	chaperone protein DnaK	68.2/4.9	1.65	0.0056
		**Cell envelope**			
1653	BRA0423	31 kDa outer-membrane immunogenic protein (“Omp31-2”)	23.2/5.2	1.45	0.00034
1874	BRA0423	31 kDa outer-membrane immunogenic protein (“Omp31-2”)	23.2/5.2	1.34	0.026
		**Transport and binding proteins**			
1415	BR0639	porin Omp2a (*omp2b*)	40.5/4.6	1.41	0.03
1410	BR0639	porin Omp2a (*omp2b*)	40.5/4.6	1.4	0.028
2176	BRA0565	bacterioferritin	18.7/4.6	1.38	0.00065
1229	BRA0655	glycerol-3-phosphate ABC transporter, periplasmic	47.2/5.4	1.33	0.0043
		**Energy metabolism**			
		***ATP***-***proton motive force interconversion***			
1019	BR1800	ATP synthase F1, gamma subunit	32.0/7.8	1.6	0.021
		***Electron transport***			
1435	BRA0893	thioredoxin	34.7/4.8	−1.34	0.0045
		***Glycolysis***/***TCA cycle***			
1145	BR1132	enolase	45.4/5.0	1.43	0.0021
		**Amino acid metabolism**			
		***Biosynthesis***			
1915	BRA0883	3-isopropylmalate dehydratase, small subunit	22.5/5.0	−1.55	0.0013
221	BR1488	carbamoyl-phosphate synthase, large subunit	126.9/5.0	−1.34	0.0098
		***Degradation***			
278	BRA0725	glycine cleavage system P protein	99.9/5.8	1.51	0.00044
		***Transport***			
1219	BRA1193	amino acid ABC transporter	44.2/5.6	1.38	0,000015
1293	BRA0953	amino acid ABC transporter, periplasmic amino acid-binding protein, putative	43.3/5.3	1.36	0.0019
1549	BR0741	amino acid ABC transporter, periplasmic amino acid binding protein	37.2/5.3	1.31	0.00014
		**Protein metabolism**			
		***Biosynthesis***			
1783	BR0455	ribosomal protein S6	17.1/8.0	1.69	0.0069
1980	BR0452	ribosomal protein L9	21.0/4.8	1.59	0.00041
		***Secretion***			
313	BR1945	preprotein translocase, SecA subunit	103.0/5.1	−1.34	0.005
		**DNA**/**RNA metabolism**			
		***Biosynthesis***			
221	BR1488	carbamoyl-phosphate synthase, large subunit	126.9/5.0	−1.34	0.0098
454	BR0837	phosphoribosylformylglycinamidine synthase II	80.0/4.8	−1.31	0.01
456	BR0837	phosphoribosylformylglycinamidine synthase II	80.0/4.8	−1.31	0.015
		***Degradation***			
689	BR2169	polyribonucleotide nucleotidyltransferase	77.7/5.0	1.55	0.0029
		**Fatty acid metabolism**			
		***Degradation***			
1881	BR1510	long-chain acyl-CoA thioester hydrolase, putative	14.25/6.6	1.67	*
		**Sugar metabolism**			
		***Transport***			
1642	BR0544	ribose ABC transporter, periplasmic D-ribose-binding	34.6/4.8	1.46	*
		**Regulation**			
1743	BR0569	transcriptional regulator, Ros/MucR family	16.10/7.8	1.73	0.021
1843	BR2159	transcriptional regulator, Cro/Cl family	15.1/9.0	1.6	*
1813	BR1502	leucine-responsive regulatory protein	17.8/6.7	1.5	0.049
		**Oxidoreduction**			
1975	BRA0708	alkyl hydroperoxide reductase C	20.6/5.0	−1.39	0.005
		**Cofactor biosynthesis**			
826	BRA0491	8-amino-7-oxononanoate synthase	40.6/7.3	1.52	0.033
		**Unknown function**			
2190	BRA0336	conserved hypothetical protein	18.4/5.0	−1.42	0. 022

^*a*^The indicated number is an arbitrary designation of the annotated spots on the 2D proteome maps [see Additional files 1 and 2].

^*b*^Open reading frame number attributed by Paulsen et al. [20].

^*c*^As annotated by Paulsen et al. [20].

^*d*^Calculated from the amino acid sequence of the translated open reading frame.

^*e*^Increase or decrease of protein concentrations after normalization of protein spot intensities from 2D-DIGE gels of *B*. *suis* recovered from a 6-weeks-starvation condition as compared to normalized protein spot intensities of corresponding spots from early stationary phase control of *B*. *suis* in TS broth.

^*f*^Statistical significance of the ratio described in^*e*^.

*indicates lack of statistical validation of the ratio described in^*e*^, but increase in protein concentration was observed in each of the three independent experiments.

The group of proteins involved in adaptation to atypical environmental conditions contains two proteins: The first one belongs to the Dps family (“DNA-binding proteins from starved cells”) (spot ID 2122 and 2146), the second was identified as “putative organic solvent tolerance” protein (spot ID 429 and 438) (Table 1). Dps-like proteins are strongly conserved among bacteria and are characterized by two major functions: Protection against damage caused by oxidative stress and adaptation to starvation [21,22]. Binding of Dps to bacterial DNA results in the formation of condensed, crystalline structures in which DNA is protected against damage or degradation [23], and Dps most likely plays a direct role in gene regulation during starvation. Dps from *M*. *smegmatis*, also increased under starvation stress conditions, and for which DNA-binding has been shown experimentally, has 52% amino acid homology to *Brucella* Dps. The “putative organic solvent tolerance” protein has been described to regulate the permeability of the outer membrane, inhibiting most likely the influx of toxic molecules [24,25]. It also participates in the biogenesis of the outer membrane [26]. Brucellae may increase the concentration of this protein under starvation stress, in order to protect themselves from toxic molecules possibly released from dead bacteria.

In *E*. *coli*, expression of the “heat shock” protein DnaK is positively controlled by the σ^32^ factor (encoded by *rpoH*), also under starvation stress [27]. In starved *B*. *suis*, DnaK (spot ID 662) showed increased concentrations whereas concentrations of the co-chaperone controlling the nucleotide and substrate binding by DnaK, GrpE (spot ID 1624), was reduced. The reduced concentrations of GrpE, may result in a lowered DnaK-activity. This may finally lead to ATP saving, which might be crucial under dormancy-like conditions. In addition, DnaK turned out to be of significance during the acute phase of *B*. *suis* infection, both for intramacrophagic replication and resistance to low pH [28].

Within the group of transcriptional regulators, one induced protein belonged to the Ros/MucR family (spot ID 1743). This regulator participates in the transcription of genes involved in the succinoglycan biosynthesis of *Sinorhizobium meliloti*, a plant symbiont closely related to *Brucella*. Succinoglycan is essential for Alfalfa colonization by *S*. *meliloti* and the installation of this symbiont [29]. In macrophage and murine models of infection, the regulator MucR has been described as a virulence factor of *B*. *melitensis*[30]. Preliminary studies on a *mucR*-mutant of *B*. *melitensis* further suggest that MucR regulates exopolysaccharide biosynthesis and genes involved in nitrogen metabolism and stress response [31].

A biological function has not yet been attributed to the induced outer membrane protein Omp31-2 (spot ID 1653 and 1874). Annotated as porin, the other outer membrane protein Omp2a (spot ID 1410 and 1415), encoded by gene *omp2b*, may participate in the uptake of molecules by *Brucella*, and the extreme starvation could act as a signal to increase porin density in the membrane. The glycerol-3-phosphate transporter UgpB (spot ID 1229) was also induced under starvation, in agreement with similar observations in *E*. *coli*[32]. Interestingly, *Brucella* UgpB is cell-surface-located and plays a role as adhesin and invasin during infection of epithelial cells [33]. Glycerol-3-phosphate is a metabolic intermediate of glycolysis and phospholipid biosynthesis, and *Brucella* may try to increase the take-up of such potential energy supplier to compensate ATP deficiency. It remains to be investigated if UgpB has a double function in brucellae and whether a nutrient stress may promote subsequent invasion of host cells. The concentration of bacterioferritin (spot ID 2176), the major actor in iron homeostasis, was also increased under starvation conditions with low levels of iron. It has been described previously that the bacterioferritin-related iron pool induces membrane proteins to adapt to low iron concentrations, confirming the central role of bacterioferritin in the iron storage of *Brucella*[34].

During starvation, two enzymes involved in leucine and glutamate biosynthesis, 3-isopropylmalate-dehydrogenase (spot ID 1915) and carbamoylphosphate synthase (spot ID 221), respectively, were repressed, indicating that the bacteria reduced their metabolic activity. In contrast, concentration of the glycine cleavage system P protein (spot ID 278) increased. This protein is part of the glycine decarboxylase multienzyme complex, also annotated as glycine cleavage system, and functions as a glycine dehydrogenase. In a signature-tagged mutagenesis screen investigating long-term survival of *B*. *abortus* in mice, the P protein was identified as a factor participating in chronic persistence of the pathogen [35]. In *M*. *tuberculosis*, the activity of glycine dehydrogenase has been found to increase 10-fold upon entry into a state of nonreplicating persistence *in vitro*[6]. Another protein of this system, GcvT, has been described thereafter as being essential in intramacrophagic survival of *B*. *suis*[3]. Since this enzyme catalyzes the step resulting in release of NH_3_, activity of the glycine decarboxylase multienzyme complex may allow starving bacteria to recycle ammonium residues from glycine metabolism for minimal biosynthetic activities required under these conditions. In addition, concentrations of several amino acid transporters increased (spot ID 1219, 1293, and 1549), which is in agreement with other studies describing their positive regulation by the stringent response allowing bacteria optimal adaptation to starvation (reviewed in [36]). In the group of factors linked to protein metabolism, two ribosomal proteins (spot ID 1783 and 1980) were starvation-induced. This seems to be contradictory to the obvious shut-down of cellular metabolism. However, in order to maximize stringent response depending on the interaction of RelA with ribosomes carrying uncharged tRNA molecules, the rate of synthesis of some ribosomal subunits may be increased. In contrast, SecA (spot ID 313), participating in protein translocation/secretion, was found in lower concentrations in starved *Brucella*, indicating an additional strategy to reduce metabolic activity and energy consumption.

In analogy to the observed repression of amino acid biosynthesis, energy-consuming *de novo* DNA and RNA biosynthesis was also reduced. RNA degradation increased, indicating a higher turnover than under control conditions and enabling bacteria to rapidly recycle the corresponding molecules. Increased degradation was also noticed for fatty acids, leading to the speculation that brucellae might use own fatty acids for minimum energy supply. Indeed, the induction of a putative long-chain acyl-CoA thioester hydrolase (spot ID 1881) has been previously observed under anaerobic denitrification, suggesting a switch to β-oxidation for energy supply under anaerobic stress conditions [14].

In the group of energy metabolism-related proteins, one single subunit of the ATP synthase (spot ID 1019) was identified as being induced under starvation conditions as compared to early stationary phase in rich medium, indicating that *Brucella* attempts to counteract obvious ATP limitation. As membrane-associated proteins are not systematically separated in 2D gel electrophoresis, the identification of only one ATP synthase subunit was conceivable. Thioredoxin (spot ID 1435) participates in NADPH-dependent formation of disulfide bonds in target proteins [37], hence consuming reduction equivalents are no longer available for electron transport and ATP synthesis. The decrease in thioredoxin under starvation stress is in agreement with the observed reduction in amino acid (and therefore protein) biosynthesis, resulting in energy saving.

A single protein involved in oxido-reduction, alkylhydroperoxide reductase C (spot ID 1975), has been identified as being down-regulated under these extreme starvation conditions. In *B*. *subtilis*, AhpC was postulated to be responsible for the detoxification of endogenous organic hydroperoxides arising from unsaturated fatty acids and from nucleic acids during growth under oxidative stress [38]. In *Brucella abortus*, AhpC is the primary detoxifier of endogenous H_2_O_2_ generated by aerobic metabolism [39]. Down-regulation of this enzyme in brucellae was therefore in accordance with a reduced oxidative bacterial metabolism during long periods of starvation with absence of noticeable growth. Spots 2172, 2207, and 1455 (see Additional file 1) were identified as being significantly regulated (p ≤0.05), but the low concentrations of these proteins in the samples did not allow their identification.

## Conclusions

The aim of this work was to gain a deeper insight into the regulative processes of *B*. *suis* to survive under extreme starvation mirroring possible living conditions of the bacteria in the host or the environment. In summary, *B*. *suis* was capable to adapt to long-term, severe nutrient deficiency by the combination of three major strategies, allowing reduction of metabolism and of energy consumption to the strict minimum necessary for survival: shortened biosynthesis of amino acids, nucleic acids and thioredoxin; degradation possibly associated with the recycling of molecules (induction of the glycine decarboxylase multienzyme complex and of a putative long-chain acyl-CoA thioester hydrolase); and reduced secretion (diminished SecA synthesis). The contribution of subcellular material of dead bacteria to the survival of adapted brucellae within the culture medium remains a matter of debate. The initial decline of the growth curve of *B*. *suis* under starvation (Figure 1) does not support primary “bacterial cannibalism” as survival strategy. Despite the fact that replacement of the culture buffer did not alter survival kinetics of the bacteria, indicating a state of persistence, it cannot be completely excluded that during the observed long-term survival, a low-level balance establishes between dividing and dying bacteria and that C- and N-sources may be available at very low concentrations. In any case, a high degree of starvation is evident from the lack of increase in the number of CFUs under these conditions. Furthermore, it is interesting to mention the capability of *B*. *abortus* to fix and assimilate CO_2_ from the atmosphere as a substitute of carbon sources of organic origin [40,41]. The 2D-DIGE experiments presented in this study, however, did not allow to answer the question whether *B*. *suis* possibly fixed CO_2_ under these experimental starvation conditions.

## Methods

### *B*. *suis* long-term survival kinetics under extreme starvation conditions

*B*. *suis* 1330 (ATCC 23444) was cultivated under shaking (160 rpm/min) to the early-stationary phase in tryptic soy (TS) broth (OD_600_ of 1–1.2), and the bacterial pellet was washed twice in phosphate-buffered saline (PBS) prior to inoculation of two series of triplicate cultures, at a concentration of 10^9^ bacteria/ml (50 ml/flask). The bacteria were cultured under shaking and aeration in a salt solution derived from *Brucella* minimal medium as described by Gerhardt and Wilson [42]. This solution was devoid of any source of carbon and nitrogen and was composed of NaCl 128 mM, K_2_HPO_4_ 57 mM, Na_2_S_2_O_3_ x 5 H_2_O 0.4 mM, MgSO_4_ x 7 H_2_O 80 μM, FeSO_4_ x 7 H_2_O 360 nM, MnSO_4_ x H_2_O 600 nM, and CaCl_2_ x 2 H_2_O 272 nM. The number of viable brucellae was determined in the beginning and every week over a period of six weeks by serial dilutions and plating onto TS agar. In one of the culture series, bacteria were washed in PBS and resuspended in fresh salt solution after three weeks before the incubation was continued.

### *B*. *suis* growth conditions and harvesting of bacteria for 2D-DIGE analysis

*B*. *suis* 1330 (ATCC 23444) was cultured either in TS broth at 37°C to an OD_600_ of 1–1.2 as reference condition, or for a period of six weeks in *Brucella* minimal medium (see above). Briefly, prior to culture in the salt solution, *B*. *suis* was cultivated under shaking (160 rpm/min) to early-stationary phase in 50 ml of TS broth (OD_600_ of 1–1.2), and the bacterial pellet was washed twice in PBS before resuspension in 500 ml of the salt solution and incubation under shaking and aeration. Three independent cultures were performed in parallel. The number of viable brucellae determined at 0, 14, 21, 28, 35 and 42 days post-inoculation by serial dilutions and plating onto TS agar was comparable to the numbers shown in Figure 1. After six weeks, the bacteria were harvested by centrifugation and washed twice in ice-cold PBS. This preparation procedure eliminated soluble proteins and membrane fragment-bound proteins of dead bacteria. Lysis of viable, starved bacteria and precipitation of total bacterial proteins was achieved using 10% trichloroacetic acid (TCA) for 1 h on ice. The proteins were washed twice with acetone and dried.

### Sample preparation

All preparations of the bacterial samples from three independent experiments were carried out at 4°C. The precipitated proteins were resuspended in sample buffer (30 mM Tris, 7 M urea, 2 M thiourea, 4% (w/v) CHAPS, pH 8.5). After sonication on ice (10 × 1 s; 60 W) and centrifugation (12,000 × *g*; 5 min) the supernatant was used for CyDye-labeling. Protein concentrations were determined by a Bradford-like protein assay (Bio-Rad Laboratories) and adjusted to 5 μg/μl. The pH of each sample was adjusted to 8.5.

### CyDye-labeling

CyDye-labeling was carried out according to manufacturer’s instructions (Amersham Pharmacia Biotech) and the labeled samples were stored at −70°C until use.

The protein samples of *B*. *suis* cultivated in the salt solution and of *B*. *suis* grown in rich TS medium were labeled with Cy3 and Cy5, respectively. Cross-labeling was performed in a single experiment. Equivalent amounts of pooled proteins obtained from both samples of *B*. *suis* were labeled with Cy2, creating the internal standard. Labeling of 1-2% of the available lysines in the protein samples using CyDye DIGE fluors does not significantly alter protein mobility in two-dimensional gel electrophoresis [43]. In addition, CyDye-labeling does not affect mass spectral analysis.

### Difference gel electrophoresis (DIGE) – Isoelectric focusing (IEF) and sodium dodecyl sulfate-polyacrylamide gel electrophoresis (SDS-PAGE)

Equal volumes of 2D sample buffer (7 M urea, 2 M thiourea, 1% DTT, 4% (w/v) CHAPS, 0.5% (v/v) Pharmalyte™ 3–10 (Amersham Pharmacia Biotech)) were added to the labeled proteins. Both *B*. *suis* samples and the internal standard were pooled and separated in one gel. A total of 150 μg protein per sample were applied to IPG strips (pH 4–7 and pH 6–11; 18 cm) for IEF and subsequent SDS-PAGE by rehydrating the IPG strips overnight at room temperature in 120 μl of the pooled samples and 350 μl rehydration buffer (8 M urea, 1% DTT, 4% (w/v) CHAPS, 1% (v/v) Pharmalyte™ 3–10).

IEF was performed using the DryStrip Focusing System (Amersham Pharmacia Biotech) at 20°C. A voltage gradient was applied (total of 40 kVh within 10 h, 50 μA/IPG strip). Prior to SDS-PAGE, the IPG strips were equilibrated in gel loading buffer for 10 min (120 mM Tris pH 6.8, 20% (v/v) glycerol, 4% (w/v) SDS, 200 mM DTT and traces of bromphenol blue). The second dimension-electrophoresis was carried out at 10°C using 12%-acrylamide gels (18 × 18 cm).

### Gel analysis

Protein spots were visualized with a Typhoon™ 9400 Series Variable Mode Imager (Amersham Pharmacia Biotech). The resulting gel images were processed using DeCyder Differential Analysis Software v5.02 (Amersham Pharmacia Biotech).

Protein spots were detected using the Differential In-gel Analysis (DIA) mode of ‘DeCyder’. The Biological Variation Analysis (BVA) mode allowed inter-gel matching on the basis of the in-gel standards (Cy2). Spot intensities were normalized to the internal standard. For each spot, averages and standard deviations of protein abundance were compared between the profiles of *B*. *suis* grown in rich medium and cultivated under starvation conditions. The Student’s t-test was applied to each set of matched spots. Significantly regulated proteins (p-value ≤ 0.05) were then identified by mass spectral analysis. To exclude non-real spots prior to MALDI-TOF analysis, the three-dimensional displays of significant spots were also checked manually.

### Protein identification by mass spectral analysis

Prior to spot-picking, 2D gels were stained with Coomassie to ensure that the majority of the unlabeled molecules of the proteins of interest were recovered for MALDI-MS analysis. Protein spots of interest were manually picked and washed three times in 50 mM (NH_4_)_2_HCO_3_. Then, gel spots were dehydrated in 100% acetonitril for 5 min. After removal of the supernatant, 1 μl protease-solution (0.05 μg/μl trypsin in 10 mM (NH_4_)_2_HCO_3_) was added and allowed to penetrate into the gel. Another 5–10 μl NH_4_HCO_3_-buffer (10 mM, in 30% acetonitril) were added to the gel plugs which were incubated overnight at 37°C for digestion. The samples were desalted in C_18_-ZipTips™ (Millipore, Bedford, MA, USA) according to manufacturer’s instructions. The desalted and concentrated peptides were eluted from the ZipTips™ on the MALDI targets with matrix solution (0.1% trifluoroacetic acid (TFA)/80% acetonitrile, equally mixed with 2,5-dihydroxybenzoic acid: 2-hydroxy-5-methoxybenzoic acid, 9:1).

For analysis of the tryptic peptides, MALDI-TOF mass spectrometry was carried out using the Voyager-DE™ STR Biospectrometry Workstation (Applied Biosystems). The spectra were acquired in a positive reflectron mode (20 kV) and collected within the mass range of 700 to 4,200 Da. The autolytic fragments of trypsin acted as internal calibrants.

The peptide mass fingerprint spectra were processed with the Data Explorer v4.9 Software (AB Sciex). The baseline was corrected, whenever necessary, with the following parameters: peak width 32, flexibility 0 and degree 0.01. To detect peaks the parameters valley to baseline, 50% centroid, an S/N threshold of 15, and a noise window width (m/z) of 1 were used. The S/N was recalculated from the cluster area and the threshold for peak detection was set to 20. No deisotoping was performed. Peak lists were filtered for monoisotopic masses and the charge state 1+. Both monoisotopic peptide masses and signal heights were used to query an in-house *Brucella suis* database using the search engine Mascot v2.1.04 (Matrix Science) in order to obtain corresponding amino acid sequences. All sequences currently available from NCBI (http://www.ncbi.nlm.nih.gov) were entered in the in-house database.

## Competing interests

The authors have declared no competing of interests.

## Authors’ contributions

SAD, HN and SK were responsible for the study design. SAD, VJM and SK analyzed and interpreted the data. SK and SAD wrote the report. VJM and HN helped to draft the manuscript. All authors read, commented and approved the final article.

## Supplementary Material

Additional file 1 **Detailed view of up-regulated proteins of *Brucella *under starvation conditions.** Description: Detailed view of the protein profiles of *B*. *suis* 1330 after six weeks under starvation conditions in a salt solution, as shown in Figure 2. Under starvation up-regulated proteins with their corresponding ID numbers are presented in **(A)** for proteins with a pI of 4–7, in **(B)** for those with a pI of 6–11. Click here for file

Additional file 2 **Detailed view of down-regulated proteins of *Brucella *under starvation conditions.** Description: Detailed view of the protein profiles of *B*. *suis* 1330 after six weeks under starvation conditions in a salt solution, as presented in Figure 3. Under starvation down-regulated proteins with their corresponding ID numbers are shown. Click here for file
