# Supplementary figures and images for: Quantitative analysis of the Brucella suis proteome reveals metabolic adaptation to long-term nutrient starvation
Source: BMC Microbiol. 2013 Sep 4;13:199. doi: 10.1186/1471-2180-13-199 (PMC3844638; doi:10.1186/1471-2180-13-199)

pl 4-7; UP

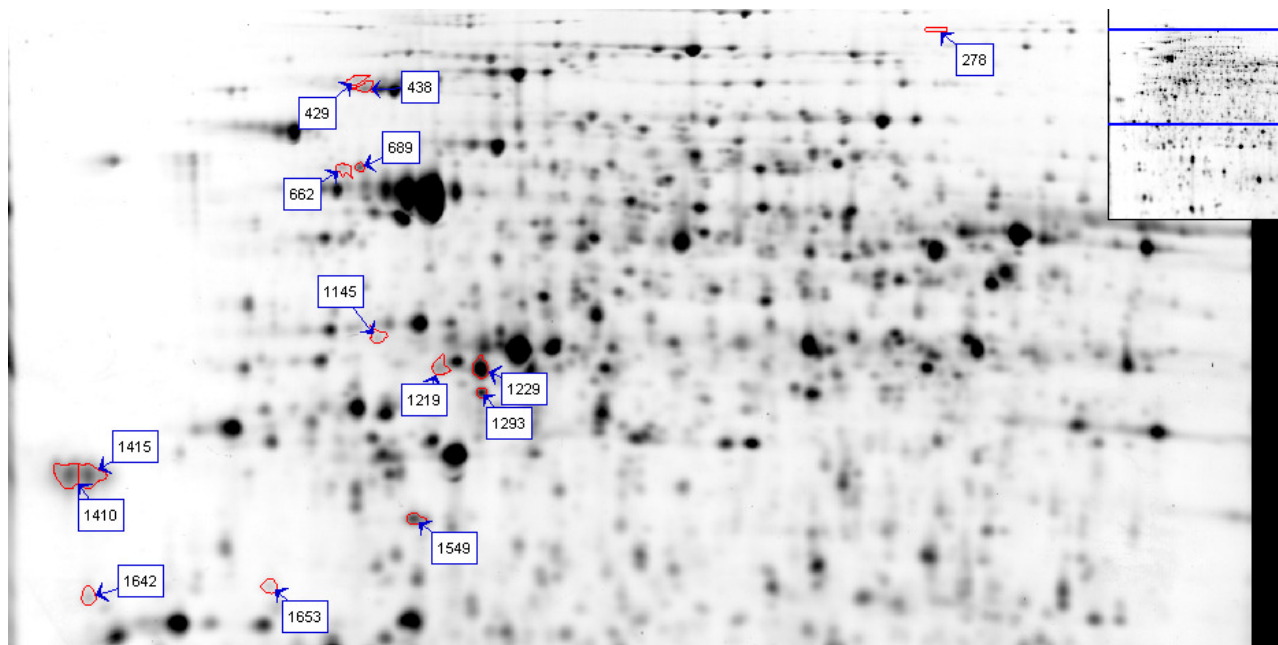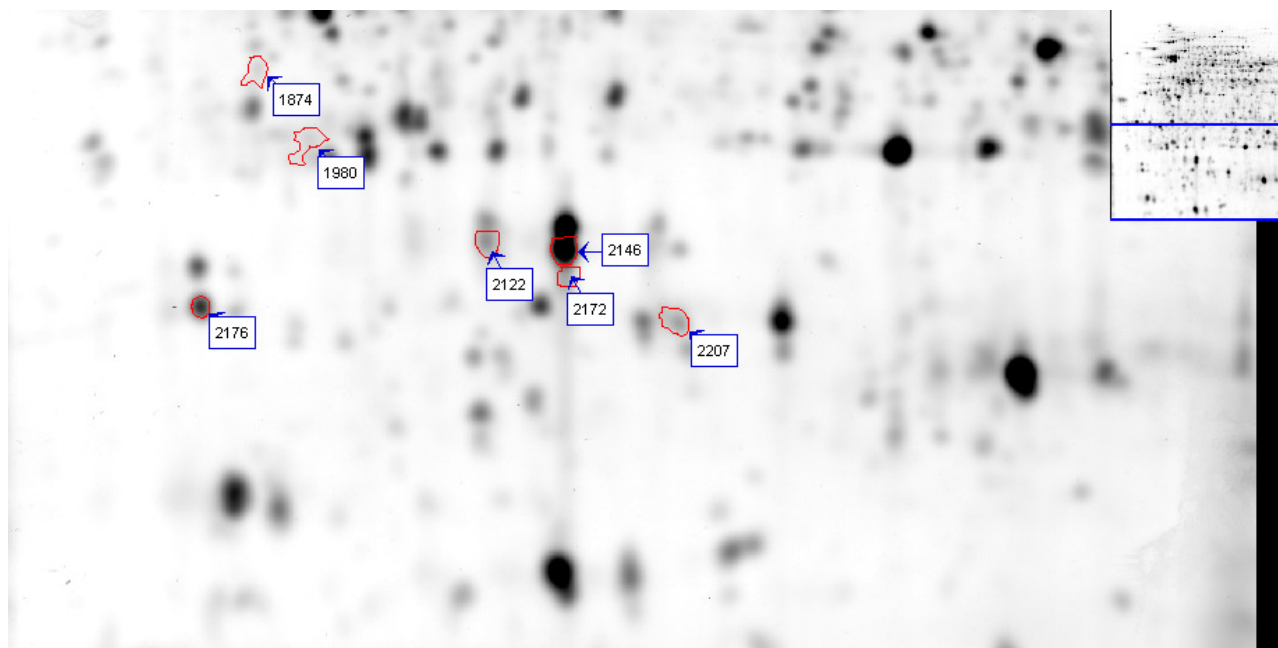

Additional file 1 A  
Supplementary to Fig. 2 A

pl 6-11; UP

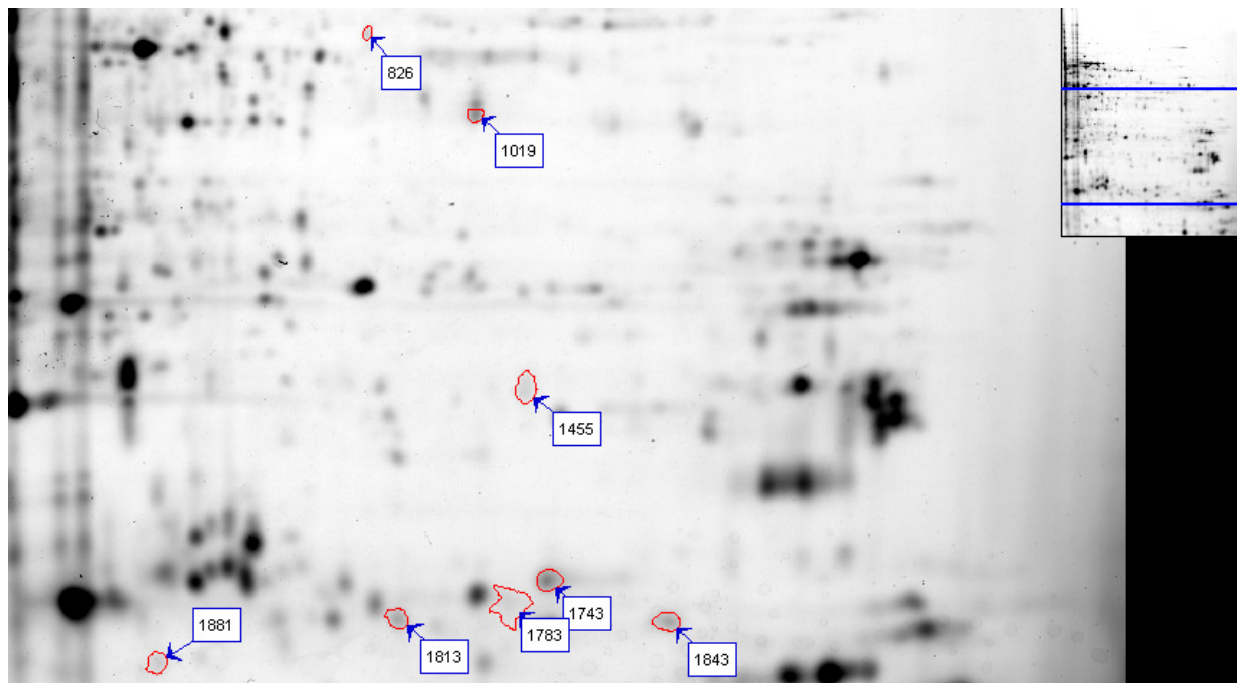

Additional file 1 B  
Supplementary to Fig. 2 B

Supplement: Additional file 1 — Detailed view of up-regulated proteins of Brucella under starvation conditions. Description: Detailed view of the protein profiles of B. suis 1330 after six weeks under starvation conditions in a salt solution, as shown in Figure 2. Under starvation up-regulated proteins with their corresponding ID numbers are presented in (A) for proteins with a pI of 4–7, in (B) for those with a pI of 6–11. [file 1471-2180-13-199-S1.pdf]

pl 4-7; DOWN

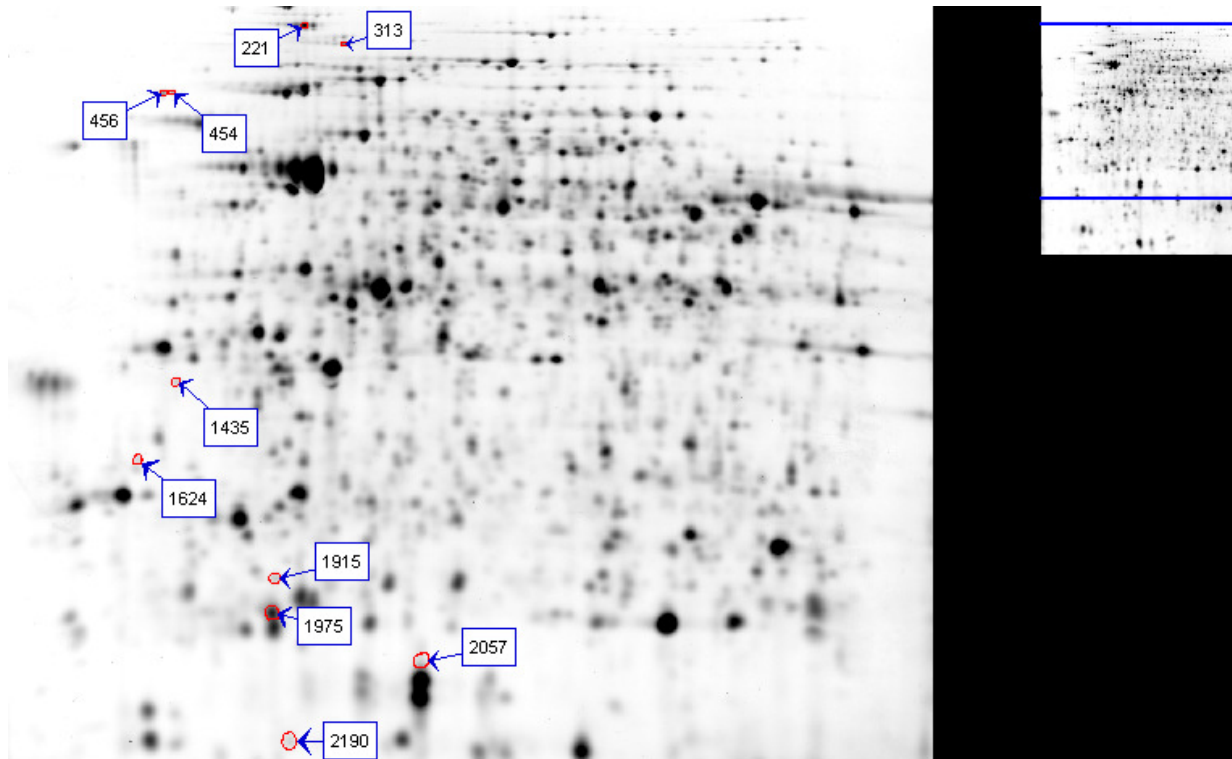

Additional file 2  
Supplementary to Fig. 3

Supplement: Additional file 2 — Detailed view of down-regulated proteins of Brucella under starvation conditions. Description: Detailed view of the protein profiles of B. suis 1330 after six weeks under starvation conditions in a salt solution, as presented in Figure 3. Under starvation down-regulated proteins with their corresponding ID numbers are shown. [file 1471-2180-13-199-S2.pdf]
